# 肿瘤合并新型冠状病毒感染的临床特征与应对策略

**DOI:** 10.3779/j.issn.1009-3419.2020.102.15

**Published:** 2020-04-20

**Authors:** 楠楠 赵, 婕 石, 丽忠 曾, 拴盈 杨

**Affiliations:** 710004 西安，西安交通大学第二附属医院 The Second Affiliated Hospital of Xi'an Jiao Tong University, Xi'an 710004, China

**Keywords:** 肿瘤, 2019新型冠状病毒（SARS-CoV-2）, 2019年新型冠状病毒疾病（COVID-19）, Tumor, SARS-CoV-2, 2019 novel coronavirus disease (COVID-19)

## Abstract

自2019年12月中旬开始，中国湖北省武汉市爆发了新型冠状病毒肺炎疫情，并迅速蔓延至中国其他省市及全球数十个国家和地区，成为国际重大突发公共卫生事件。新型冠状病毒主要通过飞沫和密切接触传播，人群普遍易感。肿瘤患者由于免疫功能受损，更是成为此次疫情中的高危人群。早期识别肿瘤并发新型冠状病毒肺炎患者，了解其分布特征，以便给予更有针对性的治疗，提高患者治愈率，更好地控制新型冠状病毒的流行和发展。本文通过对相关文献的综合分析，对肿瘤合并新型冠状病毒肺炎患者的临床特点进行了较为系统的综述，并为肿瘤患者如何应对此次疫情提出了相关建议。

## Abstract (en)

Since mid-December 2019, severe acute respiratory syndrome coronavirus 2 (SARS-CoV-2) has outbroken in Wuhan, Hubei Province, China, and spread rapidly to other provinces in China and dozens of countries and regions around the world, becoming the Public Health Emergency of International Concern (Public Health Emergency of International Concern). SARS-CoV-2 can mainly transmit by droplets or close contact, and is generally susceptible in the crowd. Tumor patients are at high risk of this pathogen because of their impaired immune function. Identifying tumor patients with 2019 novel coronavirus disease (COVID-19) early, and understanding its distribution characteristics can help to improve the cure rate of patients, and better control the epidemic and development of SARS-CoV-2 much better. With comprehensive analysis of relevant literature, this paper reviews the clinical characteristics of neoplastic patients with COVID-19, and puts forward some suggestions on how to deal with this epidemic.

2019年12月，中国湖北省武汉市出现了一种不明病原的急性呼吸系统疾病^[1]^，并在短时间内迅速蔓延至全国和世界上多个国家和地区。我国科学家很快就从患者的支气管肺泡灌洗液中分离出了一种新型冠状病毒^[2]^，世界卫生组织（World Health Organization, WHO）将其命名为严重急性呼吸综合征冠状病毒2（severe acute respiratory syndrome coronavirus 2, SARS-CoV-2），由SARS-CoV-2引起的疾病命名为2019年新型冠状病毒疾病（2019 novel coronavirus disease, COVID-19），并将其列为国际重大突发公共卫生事件。

截止到2020年3月2日，中国共确诊80, 174例COVID-19患者，累计死亡2, 915例，治愈44, 891例。由于SARS-CoV-2传播速度快，波及范围广^[3-5]^，且具有人传人的特点，可通过飞沫及密切接触传播^[3]^，因此我国将其列入《中华人民共和国传染病防治法》规定的乙类传染病，并按照甲类传染病的防控措施处理。

## 1 SARS-CoV-2的特点

冠状病毒是一种RNA病毒，外有包膜，呈球形或者椭圆形，在人类、其他哺乳动物和鸟类中广泛存在，可以造成呼吸系统、消化系统和神经系统损伤^[6]^。在几种对人类致病的冠状病毒中，大多数引起的临床症状较为轻微^[7]^，但其他两种β属冠状病毒如严重急性呼吸系统综合征冠状病毒（severe acute respiratory syndrome coronavirus, SARS-CoV）和中东呼吸系统综合征冠状病毒（Middle East respiratory syndrome coronavirus, MERS-CoV）在过去二十年里累计导致超过10, 000人患病，死亡率达10%-37%^[4]^。

SARS-CoV-2的起源仍在调查中。目前的证据认为蝙蝠可能是该病毒的主要来源，而华南海鲜市场出现的某种野生动物可能是该病毒的中间宿主^[3, 8-10]^。SARS-CoV-2是迄今为止发现的可以感染人类的冠状病毒家族中的第七位成员，属于一种新型的人感染性β属冠状病毒^[2]^，与类蝙蝠样SARS冠状病毒（bat-SL-CoVZC45和bat-SL-CoVZXC21）关系较为密切，同源性可达85%以上^[8]^。

SARS-CoV-2与SARS-CoV和MERS-CoV之间有较为显著的蛋白表达差异，其中以spike蛋白最为重要^[8, 11-13]^，可能是导致SARS-CoV-2与SARS-CoV致病性和传播能力存在差异的主要原因^[14]^。此外，Lu等^[8]^通过对SARS-CoV-2病毒的结构分析发现其可能与人类血管紧张素转换酶Ⅱ（angiotensin converting enzyme Ⅱ, ACE2）受体结合。这无疑为特异性抗SARS-CoV-2药物的研发提供了方向。

## 2 COVID-19的特点

Huang等^[4]^首先报道了41例COVID-19，其中大多数患者有武汉市华南海鲜批发市场的接触史。患者的临床症状主要包括了发热、干咳、呼吸困难、肌痛、乏力，实验室检查表现为白细胞计数正常或下降，淋巴细胞计数进行性下降，影像学多表现为病毒性肺炎样改变，多种器官可出现功能障碍（如休克、急性呼吸窘迫综合征、急性心脏损伤和急性肾脏损伤），重症和危重症患者可发生死亡，死亡率约15%。随后，Chen等^[10]^和Wang等^[15]^分别分析了99例和138例COVID-19患者，发现存在基础疾病的老年男性更容易发生SARS-CoV-2感染，临床表现更为严重，且更容易出现急性呼吸窘迫综合征（acute respiratory distress syndrome, ARDS）等并发症。

SARS-CoV-2感染的某些特征与SARS-CoV和MERS-CoV感染有一定的相似之处^[3, 16-18]^。然而，与SARS和MERS相比，COVID-19患者很少有明显的上呼吸道症状，表明SARS-CoV-2感染的靶细胞可能主要位于下呼吸道^[4]^。除此之外，COVID-19患者与MERS或SARA患者相比也较少出现胃肠道症状^[17]^，其死因主要是多器官功能障碍，其次为ARDS^[19]^，这可能与ACE2在多种器官中广泛分布有关^[20, 21]^。

## 3 肿瘤合并COVID-19的特点

近年来，我国恶性肿瘤的发病率逐年升高。根据2015年癌症流行病学统计数据，恶性肿瘤的发病率约为285.83/10万^[22]^，且大多数预后较差，严重威胁着人类的健康和生活质量。肿瘤的发病机理较为复杂，涉及多种病理生理过程，受到多种因素的影响，其中机体免疫状态的影响不可忽视。肿瘤的发生发展过程中，往往会伴有机体免疫应答的减弱。大多数的肿瘤患者经过外科手术治疗切除瘤体之后，机体的免疫功能较术前会明显的增强，但其免疫状态仍低于健康人群，且手术后的应激状态及后续的放化疗等抗癌治疗也会对免疫功能造成较大的影响。

由于恶性肿瘤患者本身免疫功能低下以及抗肿瘤治疗如放化疗或手术治疗引起的全身免疫系统抑制，导致肿瘤患者比非肿瘤患者更易感染SARS-CoV-2。Wang等^[15]^通过对138例COVID-19患者的研究发现约46.4%的患者存在一种以上的并存疾病，其中恶性肿瘤（10/138, 7.2%）是最常见的并存疾病之一。中国疾病预防控制中心^[23]^通过对44, 672例COVID-19确诊病例的流行病及临床资料进行分析后发现肿瘤患者共有107例，约占存在基础疾病总例数的0.5%，粗死亡率约为5.6%，高于总体粗死亡率（2.3%）。这提示我们肿瘤患者较易发生SARS-CoV-2感染，且预后较差。为了深入研究肿瘤患者发生COVID-19的风险以及分布特征，国家呼吸疾病临床研究中心与国家卫生健康委员会^[22]^共同合作，建立了前瞻性的COVID-19队列，收集并详细分析了来自31个省级行政区的575家医院的1, 590例患者的临床数据，首次对肿瘤合并COVID-19的患者进行了深入的统计分析。他们发现1, 590例COVID-19患者中18例有癌症病史（1%），高于中国总人口的癌症发病率。其中，最常见的肿瘤类型是肺癌（5/18, 28%）。16例COVID-19合并肿瘤的患者中4例患者（25%）在过去1个月内接受了化疗或手术治疗，其他12例患者为原发肿瘤切除后常规随访的肿瘤患者。与非肿瘤患者相比，肿瘤合并COVID-19患者年龄大（平均年龄：63.1岁*vs* 48.7岁，*P* < 0.001），多数有吸烟史（4/18 *vs* 107/1, 572, *P*=0.032），更多的患者出现呼吸急促等症状（8/17 *vs* 323/1, 377, *P*=0.039），胸部计算机断层扫描（computed tomography, CT）表现更加严重（17/18 *vs* 1, 113/1, 572, *P*=0.033），但在性别、其他症状、基础疾病或胸部X线显示的基线严重程度方面均无显著差异。最重要的是，与非肿瘤的患者相比，肿瘤患者发生严重临床事件的风险更高（*P*=0.000, 3），然而通过对肿瘤类型的进一步分析发现，肺癌患者发生严重事件的可能性与其他类型的肿瘤患者相比并不高（1/5 *vs* 8/13, *P*=0.294）。此外，在过去1个月内接受化疗或手术治疗的患者发生临床严重事件的风险要比未接受化疗或手术治疗的患者高（3/4 *vs* 6/14, *P*=0.026）。随后，研究者应用*Cox*回归模型评估发生严重事件的时间依赖性危害，发现肿瘤患者比非肿瘤患者恶化的更快，肿瘤病史是预测发生严重事件的最强风险因子。

综上所述，肿瘤患者比非肿瘤患者罹患COVID-19的风险更高，肿瘤合并COVID-19的患者除了更容易发生呼吸窘迫，其余症状和非肿瘤患者大致相似，主要包括了发热、干咳、肌痛、乏力，但影像学表现更加严重，更容易发生较为严重的并发症，年龄越大发生临床严重事件的风险越高，预后越差。提示肿瘤患者在感染SARS-CoV-2后病情发展迅速，年龄越大越容易发展为危重症，死亡率越高。因此，在疫情期间，对于近期接受放化疗或手术治疗的肿瘤合并COVID-19的老年患者，应给予更多的关注，密切关注病情变化，及时调整治疗方案。

## 4 鉴别诊断

SARS-CoV-2感染的患者主要表现为白细胞计数正常或减少，淋巴细胞计数减少，胸部CT显示双肺多发小斑片影或多发磨玻璃影（ground glass opacity, GGO），且以胸膜下分布为主^[24]^。但朱闻捷等^[25]^发现1例接受CHOP（环磷酰胺+多柔比星+长春新碱+强的松）化疗的淋巴瘤患者虽然出现了相似的改变，但并非SARS-CoV-2感染。其胸部CT显示双肺多发的磨玻璃影，以外周、胸膜为主，常规检查发现淋巴细胞减少，C反应蛋白（C reactive protein, CRP）升高，且多次SARS-CoV-2的核酸检测结果皆为阴性，最终诊断考虑为社区获得性肺炎。此外，Tian等^[26]^对2例进行手术切除的肺癌患者进行活检后发现发生SARS-CoV-2感染，但其中1例患者从未出现发热，而其血细胞计数，特别是术后第1天，显示出较高的白细胞计数以及明显的淋巴细胞减少，而另外1例患者也是在CT图像出现改变后1周才出现发热。这些提示我们，肿瘤患者由于免疫系统功能受损，受到病原菌感染后临床症状和影像学改变表现常不典型，在疾病初期常常难以辨别，给临床诊断和治疗造成了很大的困难。因此对于近期出现发热和/或咳嗽等可疑症状的恶性肿瘤患者，尤其是血液和淋巴系统的肿瘤患者，考虑诊断COVID-19之前，应注重结合病史、实验室检查、影像学改变及核酸检测等多方面综合考虑，排查其他病因，明确诊断，以免延误治疗时机。以下为COVID-19的几种常见鉴别诊断。

### 4.1 其他病原菌引起的肺炎

细菌性肺炎、其他病毒性肺炎以及传染性非典型肺炎等在病程初期出现的临床症状与SARS-CoV-2感染引起的临床症状较为相似，因此临床上需要仔细甄别。肿瘤患者发生细菌性肺炎时较多伴有脓痰，随着病程的进展或可出现较重的临床症状，一般可通过影像学检查及痰涂片或培养确诊。对于病毒、支原体、衣原体以及其他病原体引起的肺炎病情一般较轻，且白细胞常无明显的增加，影像学表现不典型。痰液分离病原体、血清免疫学检测试验对疾病的诊断有重要意义。因此，在流行季节，对肿瘤患者的病情谨慎地进行评估，进行相应的血清学检查以排除其余病原体的感染是非常有必要的。

### 4.2 肿瘤进展

COVID-19患者以发热（96%）和咳嗽（47%）为最常见的症状^[3, 10, 15]^，大多数肿瘤患者，尤其是肺癌患者，当肿瘤发生进展时可堵塞/压迫支气管或淋巴管，造成阻塞性肺炎或淋巴回流不畅等，也可出现类似症状。因此，对于肿瘤患者发热时应注意鉴别是癌性发热还是其他原因引起的发热。当肿瘤进展时，影像学上常有较明显的改变，因此应该对比影像学变化，同时结合骨扫描及生化、凝血、肿瘤标志物等实验室检查，条件允许时可进行支气管镜检查来明确诊断。

### 4.3 其他非感染性疾病

肺部的非感染性疾病主要包括结缔组织病导致的肺炎、药物相关性肺炎、弥漫性肺泡出血、特发性肺间质纤维化、放射性肺炎等，这些疾病出现的临床症状虽然与COVID-19颇为相似，但都有较为明显的临床特征，如结缔组织病常常累及全身多种器官，药物相关性肺炎常有较为明确的用药史，放射性肺炎的患者接受过放射治疗等。其诊断一般需要结合患者病史、家族史、肺功能检查、自身抗体、支气管镜检及影像学资料等进行综合分析。

## 5 诊疗及防治

针对本次COVID-19疫情中的肿瘤患者，我们提出了以下几种主要的诊疗策略。

### 5.1 加强防护

应加强对癌症患者的个人保护，进行防护知识教育和培训，加强患者的自我管理。在COVID-19流行地区，对于可接受手术治疗的患者，延后择期，短期内可根据患者情况暂辅以放化疗干预；对于病情稳定的接受放化疗治疗的肿瘤患者，应考虑推迟辅助性放化疗时间，必要时可放宽靶向药物应用条件，以减少来院时间；对于肿瘤复查的患者，应合理利用网络，进行网络复诊，必须进行门诊复诊时，应做好防护，佩戴口罩，就近选择医院进行检查，并优化复诊过程，尽量减少在医院滞留的时间，离院后及时洗手，做好个人卫生。

### 5.2 早期诊断，加强动态监测

当肿瘤患者，特别是老年患者或存在其他共患病的患者一旦出现疑似COVID-19的症状或体征，应尽早进行SARS-CoV-2核酸检测，一旦确诊感染了SARS-CoV-2，应考虑加强监测，及时治疗。目前的检测手段主要包括实时荧光RT-PCR检测或者病毒基因测序，也有报道^[27]^发现在患者的唾液及粪便中检出SARS-CoV-2病毒，但其临床意义仍有待进一步探讨。

影像学检查在协助COVID-19的诊断中有着重大意义。Song等^[24]^通过对51例COVID-19患者的CT图像分析发现，约有77%（39/51）的患者为单纯磨玻璃影，75%（38/51）的患者表现为网格状影或小叶间隔增厚；86%（44/51）的患者为双肺受累，80%（41/51）为肺后部受累，86%（44/51）为外周受累。总的来说，COVID-19的患者胸部CT主要表现为双肺多发的侧斑片状影或磨玻璃影，以外周受累为主。对于肿瘤患者而言，从发生SARS-CoV-2感染到病情严重到足以引起临床症状的时间可能会相当长，但影像学会较早地出现相应的改变，因此影像学检查在辅助诊断中起着弥足珍贵的作用。

此外，Wang等^[15]^通过对COVID-19患者的实验室相关检查进行统计分析发现COVID-19的患者大多数都有淋巴细胞减少，凝血酶原时间延长，乳酸脱氢酶升高，同时，在5例死亡的患者中，中性粒细胞计数、D-二聚体、血尿素和肌酐水平持续升高，而淋巴细胞计数持续下降（*P*=0.03）。淋巴细胞的减少，凝血的激活可能与SARS-CoV-2感染引起细胞因子的大量释放，进而出现持续性炎症反应有关，病毒感染、缺氧和休克可直接造成急性肾损伤，导致血尿素和肌酐水平升高，与患者的死亡密切相关。因此，血常规、凝血、肝肾功能等的动态观察在病程早期疾病诊断、判断病情轻重方面有着重要作用。

目前COVID-19患者体内细胞因子表达水平的改变情况备受关注。细胞因子的变化可能在判断COVID-19患者病情严重程度中起着重要作用。早期研究^[17]^发现SARS患者出现广泛的肺部损伤，这可能与血清中白介素6（interleukin-6, IL-6）、干扰素γ（interferon γ, IFN-γ）等细胞因子含量增加有关。也有报道^[28]^称MERS-CoV感染的患者出现血清中的IFN-γ、肿瘤坏死因子α（tumor necrosis factor α, TNF-α）和白介素15（interleukin-15, IL-15）等细胞因子浓度增高，提示细胞因子的变化或许在冠状病毒感染的过程中具有重要意义。因此，Huang等^[4]^对COVID-19患者体内各细胞因子表达水平的变化进行研究，指出患者感染SARS-CoV-2后也可能诱导释放大量的白介素1β（interleukin-1β, IL-1β）、IFN-γ、趋化因子IP-10（interferon inducible protein-10, IP-10）和单核细胞趋化蛋白1（monocyte chemotactic protein 1, MCP-1）等细胞因子，反馈激活Th1细胞。Th1细胞因子多为促炎细胞因子，可以促进免疫细胞增殖、活化，进一步激活免疫系统，形成体内免疫系统的“正反馈”激活。当机体发生严重感染时，细胞因子的合成、释放失衡，进而形成细胞因子风暴。与非重症监护病房（non-intensive care unit, non-ICU）的患者相比，入住重症监护病房（intensive care unit, ICU）的患者血清中粒细胞集落刺激因子（granulocyte-colony stimulating factor, G-CSF）、IP-10、MCP-1、巨噬细胞炎性蛋白1α（macrophage inflammatory protein-1α, MIP-1α）和TNF-α的浓度升高，提示发生了细胞因子风暴。因此，通过监测患者血清中细胞因子含量的变化有助于临床对患者病情的把控，提供合适的治疗方案。值得注意的是，与SARS-CoV感染不同，SARS-CoV-2感染也会增加抑制炎症的Th2细胞因子如IL-4和IL-10等的分泌^[17]^。肿瘤患者本身存在由于肿瘤负荷及后续治疗导致的Th1与Th2细胞因子失衡，在受到SARS-CoV-2感染后，可能会导致体内细胞因子的变化更加复杂，加剧体内的正负反馈失衡，导致细胞因子大量释放，体内免疫功能紊乱，进而发生多器官功能障碍。因此，肿瘤患者感染SARS-CoV-2后密切关注相关细胞因子的动态变化，可能有助于医生判断患者病情进展与严重程度，及时采取干预措施。但目前对Th1/Th2细胞因子在肿瘤合并COVID-19中的变化及具体作用机制研究尚少，还需要进行更深入的探索。

### 5.3 制定合理治疗方案

由于SARS-CoV-2与SARA-CoV和MERS-CoV之间存在的差异性，针对SARA-CoV和MERS-CoV的抗病毒药物应用于COVID-19患者时并未达到预期的临床效果^[15]^。目前临床上尚无特异性的抗SARS-CoV-2病毒的药物。因此肿瘤合并COVID-19患者的治疗主要包括以下几个方面：加强临床监测，对症治疗；治疗基础疾病，防治并发症；预防继发感染；及时进行器官功能支持，加强患者的心理疏导等。

#### 5.3.1 肿瘤治疗

国家卫健委和中医药管理局针对本次疫情期间手术治疗的建议为非急诊手术延后择期。同样地，对于可进行手术切除治疗的肿瘤合并COVID-19患者，应优先治疗SARS-CoV-2感染，待传染性降低之后再进行择期手术。对于接受放化疗的患者，应待SARS-CoV-2感染完全恢复后再继续进行后续治疗。

#### 5.3.2 一般治疗

注意休息，加强营养，监测生命体征的变化，监测血尿常规、凝血、生化、血气及影像学改变，必要时监测细胞因子的即时变化。

#### 5.3.3 抗病毒治疗

目前尚无有效的抗病毒药物。目前正在试用的抗病毒药物包括α-干扰素、利托那韦、利巴韦林、瑞德西韦等，但尚无明确的证据表明这些药物对SARS-CoV-2有特异性的抗病毒作用。

#### 5.3.4 抗菌素应用

研究认为抗菌素治疗对COVID-19患者的治疗并无明显疗效。所以应避免盲目或不恰当使用抗菌药物，尤其是广谱抗菌药。对于病程较长的肿瘤患者，若出现咳脓痰、CRP升高应高度警惕继发性细菌感染，根据药敏实验，选择合适的抗菌素。

#### 5.3.5 呼吸支持

由于肿瘤合并COVID-19的患者较易发生严重临床事件，所以应注意监测患者的血氧饱和度，及时给予呼吸支持治疗。对于危重症患者，给予鼻导管或面罩吸氧，快速纠正患者的低氧血症及水电解质失衡状态。若低氧血症状态无法纠正，可考虑给予高流量鼻导管氧疗或无创呼吸机治疗。若仍未明显缓解或进一步恶化，应根据条件选择给予小潮气量低吸气压力的有创呼吸机治疗或者气管插管。

#### 5.3.6 心理疏导

肿瘤患者由于本身病情以及疫情的影响，更容易产生紧张、焦虑等不良情绪，导致机体长期处于应激状态，影响其身心健康。因此，临床上应注重对患者进行及时的心理疏导，减轻对自身病情的恐惧和忧虑，树立积极的抗击病魔的心态，必要时可在精神心理科指导下给予适当的药物干预。

#### 5.3.7 中医药治疗

中医有着悠久的历史，蕴含着中华民族几千年的经验总结。很多中医论著中记录的经典方剂对此次疫情中患者治疗方案的制定具有重要的借鉴意义。但由于中药成分的复杂性及不同个体间的差异性，患者切勿自行服用中药治疗，应在临床医师指导下，结合患者自身病情，并参照国家新型冠状病毒肺炎诊治方案进行合理有效的中医中药治疗。

#### 5.3.8 其他治疗方式

目前COVID-19的治疗还包括激素、肠道微生态调节剂、血液净化等，可作为危重症患者的辅助治疗手段，若条件允许时还可采用恢复期血浆治疗。

## 6 问题与展望

尽管目前我国的COVID-19疫情较前有所好转，但在国际上的总体形势依然较为严峻，我国仍应该继续保持警惕。由于目前针对SARS-CoV-2感染并无统一的治疗方法，相关疫苗也尚在研制中，国际上尚无相应的指南可以指导临床治疗。而且对于肿瘤患者来说，其罹患COVID-19的风险更高，加之肿瘤患者常伴随有严重的基础性疾病，在感染SARS-CoV-2后发生严重临床事件的风险更高，对药物反应差，死亡率更高，临床上应予以更多关注。因此，肿瘤患者应更注重日常生活中的个人防护，减少患病风险；疑诊患者及时上报，严格遵守隔离制度，减小传播风险；临床上依据患者的不同情况制定更合理的治疗方案，提高临床治疗疗效，改善患者预后，最大程度地保护患者生命安全。
